# A Novel Reporter Rat Strain That Conditionally Expresses the Bright Red Fluorescent Protein tdTomato

**DOI:** 10.1371/journal.pone.0155687

**Published:** 2016-05-19

**Authors:** Hiroyuki Igarashi, Kyo Koizumi, Ryosuke Kaneko, Keiko Ikeda, Ryo Egawa, Yuchio Yanagawa, Shin-ichi Muramatsu, Hiroshi Onimaru, Toru Ishizuka, Hiromu Yawo

**Affiliations:** 1 Department of Physiology and Pharmacology, Tohoku University Graduate School of Medicine, Sendai, Miyagi, Japan; 2 Japan Society for the Promotion of Science, Chiyoda-ku, Tokyo, Japan; 3 Tohoku University Division for Interdisciplinary Advanced Research and Education, Sendai, Miyagi, Japan; 4 Department of Developmental Biology and Neuroscience, Tohoku University Graduate School of Life Sciences, Sendai, Miyagi, Japan; 5 Department of Genetic and Behavioral Neuroscience, Gunma University Graduate School of Medicine, Maebashi, Gunma, Japan; 6 Bioresource center, Gunma University Graduate School of Medicine, Maebashi, Gunma, Japan; 7 Division of Biology, Hyogo College of Medicine, Nishinomiya, Hyogo, Japan; 8 Division of Neurology, Department of Medicine, Jichi Medical University, Shimotsuke, Tochigi, Japan; 9 Center for Gene & Cell Therapy, The Institute of Medical Science, The University of Tokyo, Tokyo, Japan; 10 Department of Physiology, Showa University School of Medicine, Shinagawa, Tokyo, Japan; Infectious Disease Research Institute, UNITED STATES

## Abstract

Despite the strength of the Cre/loxP recombination system in animal models, its application in rats trails that in mice because of the lack of relevant reporter strains. Here, we generated a floxed STOP tdTomato rat that conditionally expresses a red fluorescent protein variant (tdTomato) in the presence of exogenous Cre recombinase. The tdTomato signal vividly visualizes neurons including their projection fibers and spines without any histological enhancement. In addition, a transgenic rat line (FLAME) that ubiquitously expresses tdTomato was successfully established by injecting intracytoplasmic *Cre* mRNA into fertilized ova. Our rat reporter system will facilitate connectome studies as well as the visualization of the fine structures of genetically identified cells for long periods both *in vivo* and *ex vivo*. Furthermore, FLAME is an ideal model for organ transplantation research owing to improved traceability of cells/tissues.

## Introduction

The spatiotemporal restriction of genetic manipulation has helped elucidate the causal relationships between specific genes and their corresponding phenotypes in model animals. For example, simple suppression or elimination of a gene is often accompanied by homozygous lethality during the embryonic and early infantile period [1]. Even in nonlethal cases, the extensive loss or overexpression of a gene inevitably induces compensation at the molecular, anatomical, or physiological level with or without obvious abnormalities such as behavioral disorders, carcinogenicity, and physical and mental dysfunctions. The Cre/loxP recombination system is a widely used strategy to manipulate genes of interest under spatiotemporal regulation [2,3]. The conditional knockout of a target gene is widely used in mouse models, with various driver strains that express Cre recombinase from the P1 bacteriophage or its derivative under a certain promoter as well as reporter strains that retain the loxP-flanked (i.e., “floxed”) target gene. Similarly, an exogenous gene is expected to be spatiotemporally expressed using reporter animals that have the designer gene in floxed-stop or floxed-inverse (FLEX) cassettes. It is possible to determine whether a gene of interest is necessary or sufficient for an animal’s physiology and behavior by suppressing or enhancing that gene in a spatiotemporally precise manner. The Cre-loxP–dependent expression of certain genetic markers in a specific subset of cells is also a powerful tool for analyzing cell lineages and tracing transplanted cells *in vivo*.

Previous neuroscience studies using Cre-loxP technology in mice have revealed that individual groups of neurons in neural pathways underlie various basic brain functions and play significant roles in neural development [4,5]. Although rats offer potential advantages over mice for investigating higher-order brain functions because of their larger body size and superior ability to accomplish complex behavioral tasks [6], there are relatively few well-characterized *Cre* driver rat strains; this is in part because of the lack of relevant reporter strains that enable the convenient screening of driver strains.

The use of bacterial artificial chromosomes (BACs) has become an attractive method for establishing transgenic (Tg) animals, because their size (>100 kb) ensures a high likelihood of maintaining all positive and negative regulatory elements that normally control the expression of the transgene [7,8]. In addition, transgenes containing large regions of 5' and 3' flanking sequences help minimize the effects of surrounding genomic elements at the integration site, which might alter expression [9]. Ubiquitous reporter gene expression in rodent models can be achieved by utilizing a BAC clone containing ROSA26 locus [10], which directs generalized expression [11]. Here, we generated reporter rats that express tdTomato, a fluorescent protein variant, under control of a ubiquitous promotor, cytomegalovirus immediate early enhancer/chicken β-actin/rabbit β-globin hybrid promoter (CAG promoter) [12] conditionally in the presence of exogenous Cre recombinase. The brightness of tdTomato emission at relatively long wavelengths makes it ideal for live-animal imaging studies, because the absorption coefficient of tissue is reduced for light >590 nm [13]. This new rat line (NBRP-0734) will facilitate the screening of driver strains and marking of cells under specific promotors, enabling the tracing of their lineages and fates as well as analyzing their morphology *in vivo*.

## Materials and Methods

### Construction of ROSA26/CAG-floxed STOP-tdTomato BAC

The ROSA26/CAG-floxed STOP-tdTomato BAC was generated by the BAC recombineering method [14] using a BAC clone containing the mouse ROSA26 locus. Briefly, a BAC clone (RP23-244D9) from a C57BL/6J mouse genomic BAC library (BACPAC Resource Center, Oakland, CA, USA) spanning from ~183 kb upstream of ROSA26 exon 1 to ~34 kb downstream of ROSA26 exon 3 (total size: ~226 kb) was used. To modify the Rosa26 locus, a cassette containing the following components was constructed: a CAG promoter derived from pCAGGS [12], loxP, FRT-flanked Kan/Neo cassette (from J. Takeda, Osaka University), 4× poly(A) (from M. Yamamoto, Gunma University), tdTomato cDNA [15], and SV40 poly(A). Through lambda red protein-mediated homologous recombination in *E*. *coli* EL250 [16], the abovementioned cassette flanked by the homologous fragments was inserted into the ROSA26 BAC clone at the desired location. The following primers were used to generate 5′ and 3′ homology regions: R5-FS, gtcgaCGTCGTCTGATTGGCTCTC and R5-RX, ctcgaGACTGGAGTTGCAGATCAC for the 5′ homology region; R3-FS, gtcgACAGTGTCGCGAGTTAGA and R3-RX, ctcgagCACCTGAACTTTGCATTCC for the 3′ homology region. Recombinants were identified by screening for kanamycin resistance followed by PCR analysis. The following primers were used to check the integrity of the recombination: R5upF2, CGTCTCGTCGCTGATTGGCTTC and CAG-R2, CCGTAAATAGTCCACCCATTGACG for the 5′ site; G/RFPtail-F, CATGGACGAGCTGTACAAG and R3dwnR2, ATGCCATGAGTCAAGCCAG for the 3′ site. A loxP site in the pBACe3.6 backbone was removed, and the PI-SceI site was inserted using the PIsac-mLK cassette by the BAC recombineering method.

### Generation of ROSA26/CAG-floxed STOP-tdTomato BAC Tg rats

Recombined ROSA26/CAG-floxed STOP-tdTomato BACs were linearized by PI-SceI digestion and subsequently gel purified [8]. The purified BAC fragment was microinjected into pronuclei of Long–Evans (LE) rat oocytes as described previously [16]. Among the 238 oocytes injected, 210 could be transferred into pseudopregnant female rats; 51 rats were born from them. Three of 51 weaned pups were identified as the BAC Tg founders carrying ROSA26/CAG-floxed STOP-tdTomato gene, and one of them was lined herein. The probability of successful Tg generation was 1.4% (Weaned pups number (n = 3) divided by the number of transferred oocytes (n = 210)). The rats were maintained on an LE genetic background (Institute for Animal Reproduction, Ibaraki, Japan) and distributed from the National BioResource Project for the Rat (NBRP) in Japan under strain name LE-Tg(Gt(ROSA)26SorCAG-tdTomato)24Jfhy (No. 0734).

### Generation of FLAME

The STOP cassette, which consisted of a FRT-flanked Kan/Neo cassette and 4× poly(A), was removed from the ROSA26/CAG-floxed STOP-tdTomato BAC Tg rat genome by Cre-mediated excision. The capped and polyadenylated mRNA encoding Cre recombinase was synthesized by *in vitro* transcription using mMESSAGE mMACHINE T7 ULTRA Transcription Kit (ThermoFisher Scientific) and injected into the cytosol of each oocyte obtained from wild-type LE females mated with ROSA26/CAG-floxed STOP-tdTomato BAC Tg males. STOP excision was examined by PCR using genome DNA from the tail and the following primer sets: pCX1624F, CTAGAGCCTCTGCTAACC and PGK-R, GACGTGCTACTTCCATTTGTCAC for the STOP-remaining allele (PCR product: 497 bp); pCX1624F, CTAGAGCCTCTGCTAACC and Tomato+41R, CGCATGAACTCTTTGATGACCTC for the STOP-deleted allele (PCR product: 217 bp). The rats harboring the STOP-deleted R26tdT allele, designated FLAME, were maintained on an LE genetic background. Two strains were deposited in the NBRP as LE-Tg(Gt(Rosa)26Sor-CAG-tdTomato)9Jfhy (No. 0789) and LE-Tg(Gt(Rosa)26Sor-CAG-tdTomato)14Jfhy (No. 0790).

Through the characterization process, movie and overview images of FLAME pups were taken by video camera (HDR-CX630V, SONY, Japan) with the 510 nm excitation light illuminator (Epi-Green Pro, Relyon, Japan) and 590 nm longpass filter (Relyon, Japan).

All animal experiments were approved by the Tohoku University Committee for Animal Experiments (Approval No. 2014LsA-023) and were carried out in accordance with the Guidelines for Animal Experiments and Related Activities of Tohoku University as well as the guiding principles of the Physiological Society of Japan and the National institutes of health (NIH), USA. The number of animals in this study was kept to a minimum. To minimize their suffering before decapitation, both adult and P0 rats were deeply anesthetized with isoflurane in a glass bottle until nociceptive reflexes induced by tail pinch were completely abolished. Alternatively, the P0 pups were killed by the overdose inhalation of isoflurane. Animals had access to food and water ad libitum and were kept under a 12-hour light-dark cycle. Their health and behavior were weekly checked by the authors and remained sound throughout experiments.

### Identification of transgene-inserted locus

The inserted locus of the R26tdT transgene in the ROSA26/CAG-floxed STOP-tdTomato BAC Tg rat genome was identified by Straight Walk, an improved ligation-mediated PCR method [17].

### Detection of transgene recombination

Genomic DNA was extracted either by the standard method [18] or simple alkali isolation. In the alkali extraction, the animal tissue was immersed in the 50 mM NaOH and vortexed for 10 sec at room temperature. After heating for 10 min at 95°C, the solution was neutralized by 1 M Tris-HCl and centrifuged at 12,000 rpm for 10 min. Only the supernatant was used for subsequent later PCR experiments. Genomic DNA was used as the template for PCR reactions for detecting transgene. For genotyping, PCR fragments were amplified at 94°C for 2 min, then 98°C for 10 sec, 68°C for 60 sec for 30 cycles using primer set (5′-CTATGACTGGGCACAACAGACAAT-3′ & 5′-AACTCGTCAAGAAGGCGATAGAAG-3′) detecting the Neo resistance gene coding region. To evaluate the effectiveness of recombination, each allele with/without STOP sequence was PCR-amplified using either set of primers: 5'-CTAGAGCCTCTGCTAACC-3' & 5′-GACGTGCTACTTCCATTTGTCAC-3′, or 5′-CTAGAGCCTCTGCTAACC-3′ & 5′-CGCATGAACTCTTTGATGACCTC-3′. The PCR fragments were amplified at 95°C for 3 min, then 95°C for 30 sec, 60°C for 30 sec, and 72°C for 1 min for 35 cycles, then 72°C for 7 min.

### Viral expression of Cre recombinase

We used AAV2, which consists of the vector containing inverted terminal repeats (ITR)-cytomegalovirus (CMV) promoter-Cre recombinase gene-SV40poly(A)-ITR, at the concentration of 4.5 × 10^11^ vector genomes/mL as described previously [19]. For the virus-mediated expression of Cre recombinase, tdTomato reporter rats at 21–40 days old were anesthetized by ketamine/xylazine (50 and 10 mg/kg, respectively, intramuscularly) and placed in a stereotaxic frame. The scalp was incised, and a 1–2-mm craniotomy was made over each injection coordinate point. The virus used was Adeno-associated virus encoding *Cre* (AAV2-Cre) with the CMV promoter (4.5 × 10^11^ vector genomes/mL) as described previously. Virus (1.5 μL) was pressure injected at each spot. The scalp was closed by a cyanoacrylate adhesive (Aron alpha A, Daiichi Sankyo, Tokyo, Japan). The expression of Cre recombinase was identified using immunohistochemistry of Alexa Fluor 488 as described in the following section.

### Immunohistochemistry

Adult rat brains were resected three weeks after AAV2-Cre injection and promptly sectioned at 250 μm using a vibratome (VT 1000S, Leica). Tissue slices were fixed with 4% paraformaldehyde, reacted with anti-Cre antibody (1:500, MAB3120, Millipore) and anti-DsRed antibody (1:500, #632496, Clontech), followed by secondary antibodies conjugated with Alexa Fluor 488 (1:200, A11006, Molecular Probes, Eugene, OR, USA) and Alexa Fluor 546 (1:200, Z25004, Molecular Probes, Eugene, OR, USA), respectively. Immunoreactivity was assessed by fluorescent microscopy (Axiovert200, Carl Zeiss) or confocal microscopy (LSM510META, Carl Zeiss) or FV1200, Olympus). For the detection of each fluorescence substrate, following optical combinations were used: a 488 nm argon laser and 525 ± 25 nm bandpass filter for Alexa Fluor 488, a 543 nm HeNe laser and 590 ± 25 nm bandpass filter for Alexa Fluor 546.

To evaluate the efficiency of *in vivo* recombination, brain slices (16 μm) were prepared from AAV2-Cre injected brain tissue and immunostained against Cre and tdTomato. In each slice, the immunolebeled cells were counted in an unintentional visual field (0.088 mm^2^) selected from a brain slice of the AAV-injected region.

### Primary culture of fibroblast

Approximately 0.5 cm^2^ tissue fragments were dissected from the tip of the tail of Tg rats and transferred into a tissue culture dishes containing Dulbecco's modified Eagle's medium (Wako, cat. no. 043–30085) supplemented with 10% fetal bovine serum (Invitrogen, cat. no. 26140–111). While the tiny pieces of the tissue were incubated at 37°C for 2–5 days, the migrated fibroblasts were collected for the latter experiments after they reach 70–80% confluence [20]. The fibroblasts were then transfected with expression plasmids (Lipofectamine^®^ 2000, Invitrogen) according to the manufacturer's instructions.

Ten fields of interest were chosen unintentionally from each well (Nunc, cat. no. 176740). Double-blind counting was applied to detect fibroblasts which were positive for both tdTomato and AcGFP.

### Brain tissue clearing

For tissue clearing, we used CUBIC reagent-1 [21] containing 25% (w/w) urea (35904–45, Nacalai Tesque Inc., Japan), 25% (w/w) N,N,N′,N′-tetrakis(2-hydroxypropyl) ethylenediamine (T0781, Tokyo Chemical Industry Co., Ltd., Japan), and 15% (w/w) polyethylene glycol mono-*p*-isooctylphenyl ether/Triton X-100 (25987–85, Nacalai Tesque Inc., Japan). Fixed brain tissues were sliced into 1-mm-thick coronal sections in phosphate-buffered saline using a microtome (CM 3050S, Leica Microsystems, Wetzlar, Germany), immersed in CUBIC reagent-1 at room temperature for 2 days with gentle shaking, and then served for imaging.

### Imaging

Cleared brain slices within CUBIC reagent-1 were sandwiched by glass coverslips with ~1.5-mm-thick spacers and imaged under a two-photon microscopy system (A1R MP+, Nikon, Tokyo, Japan) equipped with a 16× water-immersion objective (NA 0.8) in the following optical combinations: 1020-nm laser for excitation and 629 ± 56-nm bandpass filter for tdTomato fluorescence.

Z-stack images were converted to 16-bit TIFF data with ImageJ (http://rsb.info.nih.gov/ij/) and projected maximally three-dimensionally using Zen software (Carl Zeiss, Oberkochen, Germany).

### *In utero* electroporation

A timed-pregnant wild-type LE female rat (17–18 days post coitum) mated with a tdTomato reporter male rat was anesthetized with ketamine/xylazine (50 and 10 mg/kg, respectively, intramuscularly). The uterus was lifted from the abdominal cavity, and the embryos were visualized. A glass pipette a 20–30-μm-diameter tip was introduced into the left lateral cerebral ventricle, and injected with ~2 μL DNA solution containing *AcGFP-NCre* plasmid (0.42 g/L) and 0.05% Fast Green. The capillary was removed, and a forceps with round electrodes was placed on either side of the head outside the uterine wall. Five 50-V square pulses (50 ms duration, 950 ms interval) were delivered via a square pulse electroporator (CUY21EDIT II, BEX Co., Ltd., Tokyo, Japan). The uterus was returned to the abdominal cavity, and the abdominal wall and skin were sutured. Pups were born by natural delivery. Only pups exhibiting strong red fluorescence in the brain at postnatal day 0 (P0) were used in subsequent immunohistochemistry experiments.

### Statistical analysis

All data in the text and figures are expressed as mean ± SEM and were evaluated for statistical significance with the method noted in the text or figure legends. It was judged as statistically insignificant when P > 0.05.

## Results

### Cre-dependent expression of tdTomato

**Fig 1:**
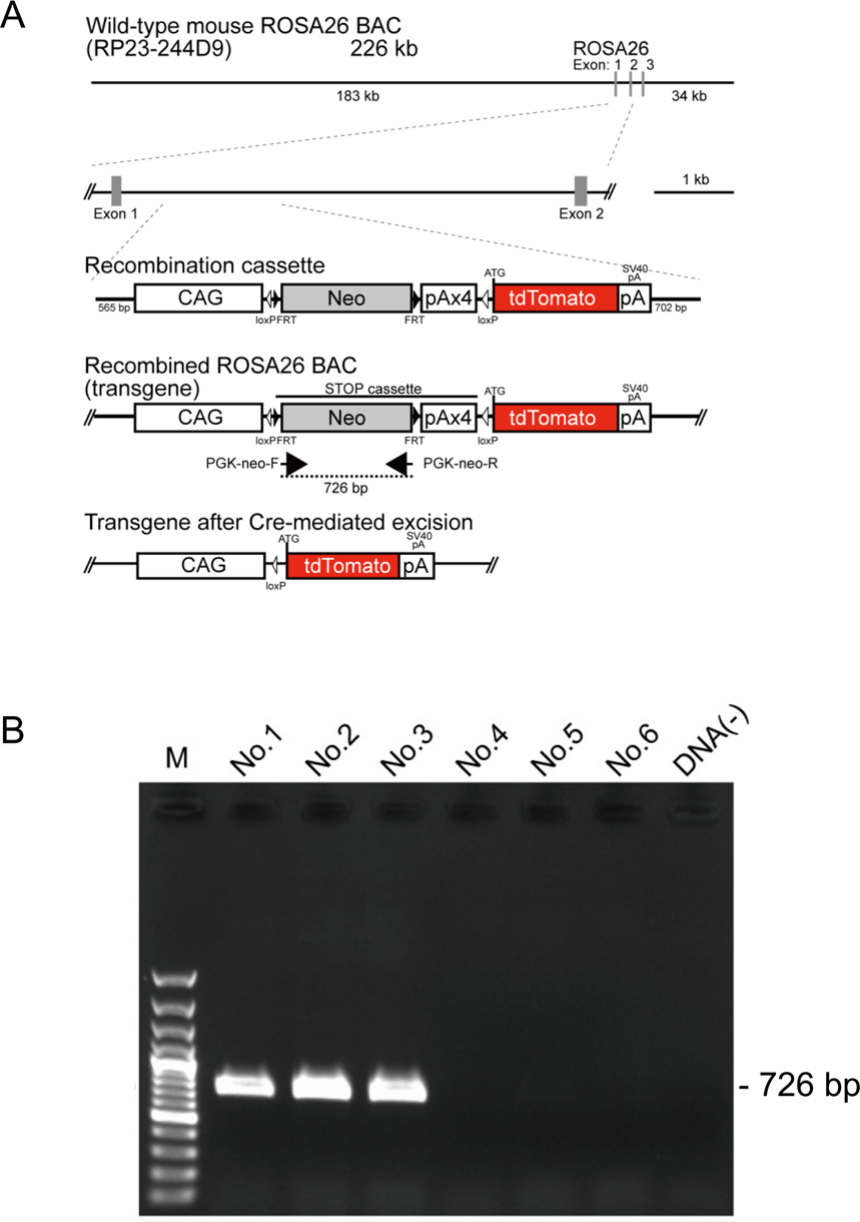
Generation and detection of tdTomato BAC reporter rats. (A) The transgene, which was inserted into the mouse ROSA26 BAC clone, comprised the CAG promoter, a cassette for the neomycin resistance gene with four STOP signal repeats (pA) flanked by loxP sites, and a sequence containing the tdTomato open reading frame. After Cre-mediated recombination, tdTomato expression was under the regulation of the generic CAG promoter. (B) PCR analysis of genomic DNA. The 726-bp band, which is recognized by the designed primer sets targeting the PGK-neo cassette located in between two loxP sequences, was present in the tdTomato reporter rats but not in the wild-type rats.

We established one reporter line, NBRP-0734 from three BAC Tg founders, carrying the tdTomato gene in the downstream of a floxed STOP cassette (Fig 1A). The gene was controlled under CAG promoter at the ROSA26 site of mouse BAC containing the ROSA26 gene [22]. After Cre/loxP site-specific excision of the STOP cassette containing PGK-neomycin–resistant gene and four repeats of polyadenylation (STOP) sequence, tdTomato was expressed under the ubiquitously active CAG promotor. PCR analysis confirmed that Tg rats possessed a PGK-neo cassette. Those with the 726-bp band were considered floxed STOP-positive and used for further experiments (Fig 1B). In addition, the insertion site of the purified BAC fragment was identified to be between loci 13,835,107 and 13,835,780 on chromosome 17 using Straight Walk [17].

### Virus-mediated reporter gene expression

**Fig 2:**
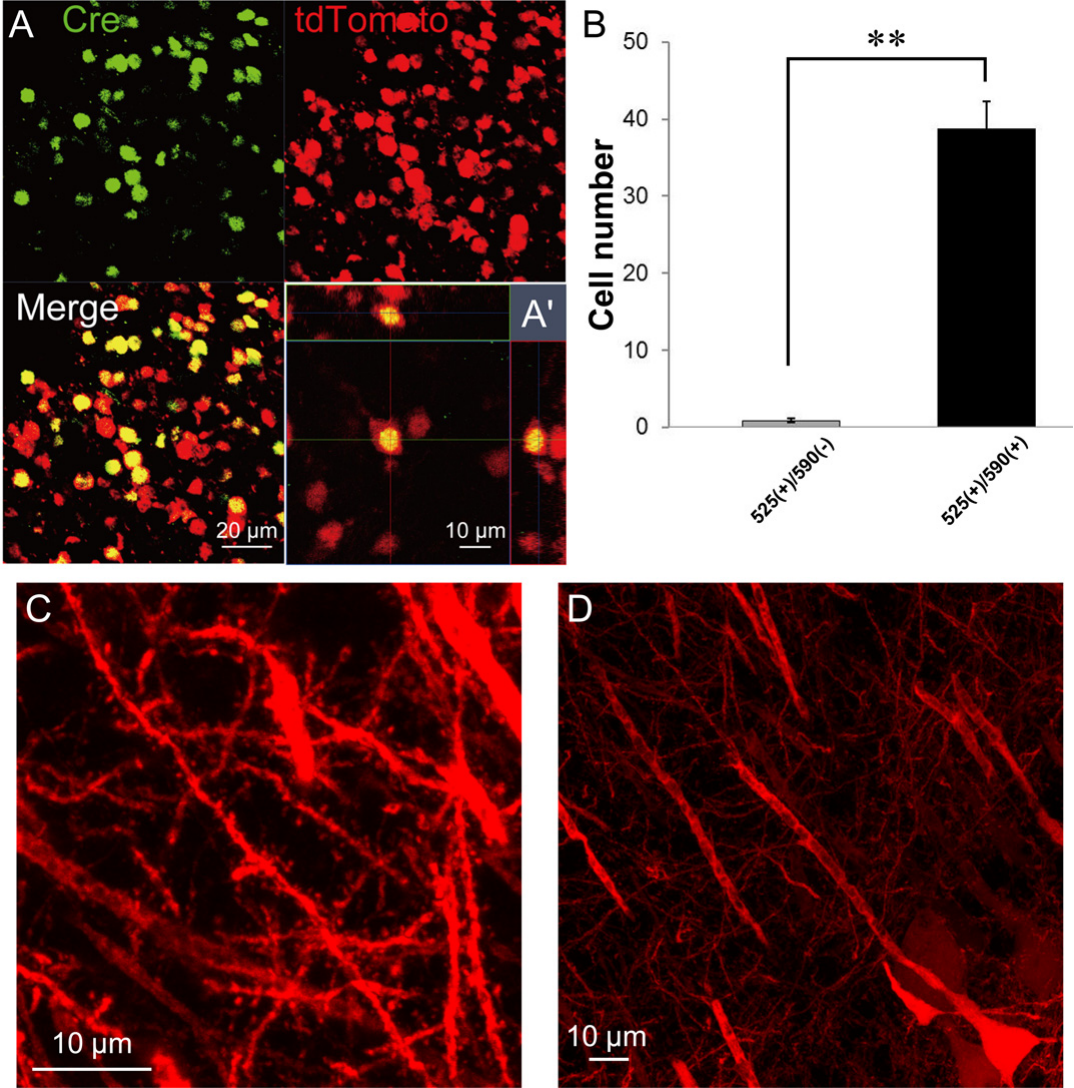
AAV2-Cre-mediated reporter gene expression in the brain. (A) Co-localization of Cre-immunoreactive nuclei (green) and tdTomato immunoreactivity (red) in the same cells [merged image and the 3D reconstruction image (A′)]. (B) Quantitative analysis for the *in vivo* recombination efficiency. 525(+)/590(-) or 525(+)/590(+) cells were counted from immunostained brain slices (n = 12). **, *p* < 0.001, Mann-Whitney *U*-test. (C) Morphology of neurons in the hippocampal CA1 region. The projecting axons were traceable to fine branches (fixed 20-μm slice without enhancement). (D) Enlarged image of CA1 dendrites. Note that fine structures such as spines can be visualized clearly.

To examine tdTomato expression following the complete excision of the STOP cassette stuffer, AAV2-Cre virus vectors containing *Cre* was injected into the striatum (*n* = 8), hippocampus (*n* = 7), and cerebellum (*n* = 2) of heterozygous rats obtained by crossing male NBRP-0734 with wild-type female LE rats. In all rats, bright red fluorescence was detected only in regions around the injection site. The Cre-immunoreactive nuclei (Alexa Fluor 488) and tdTomato immunoreactivity (Alexa Fluor 546) were colocalized within the same cells (Fig 2A). To evaluate the efficiency of *in vivo* recombination each number of cells was counted which were 525 nm (center emission wavelength for Alexa Fluor 488)-positive but 590 nm (center emission wavelength for Alexa Fluor 546)-negative (525(+)/590(-)) or were both 525 nm- and 590 nm-positive (525(+)/590(+)) in the brain slices (Fig 2B). The number of 525(+)/590(+) cells was 39 ± 3.5 cells/0.88 mm^2^ whereas that of 525(+)/590(-) cells was 0.83 ± 0.27 cells/0.88 mm^2^ with significant difference (P < 0.001, Mann-Whitney *U*-test, *n* = 12). Therefore, almost all the cells expressing Cre underwent successful recombination.

**Fig 3:**
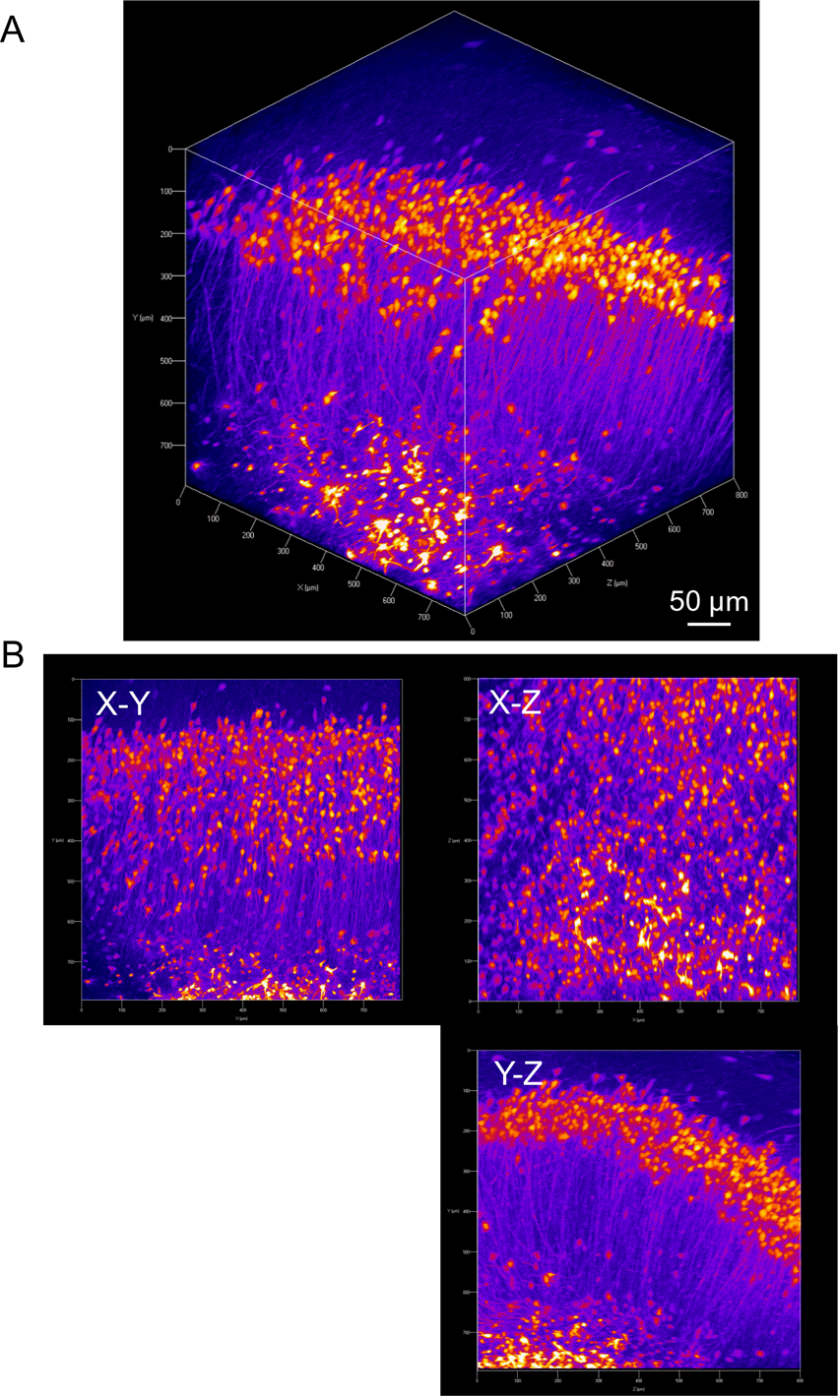
Three-dimensional imaging of hippocampal CA1 after tissue clearing. (A) Three-dimensional reconstruction of the CA1 region of the hippocampus, which was treated with CUBIC reagent 1 for 2 days and imaged under two-photon microscopy. The laminar structure of pyramidal cells was obvious. Axons and dendrites were brightly labeled by tdTomato and easily traced throughout the tissue. (B) X–Y, X–Z, and Y–Z planar sections of the image shown in (A).

The tdTomato fluorescence signals were conserved even after paraformaldehyde fixation, and fine structures such as spines were still resolvable without immunohistochemical enhancement (Fig 2C and 2D). Under close inspection, tdTomato was expressed mostly in neurons as presumed from the neuron-targeting serotype of the virus. Fluorescence from projection fibers was even visible without histological enhancement. After tissue clearing of the hippocampus, two-photon imaging clearly visualized a set of pyramidal cells in the CA1 region, demonstrating further ability to trace neurons deep in the tissue (Fig 3, S1 and S2 Video). As a control, phosphate-buffered saline was delivered into the brains of NBPR-0734 heterozygotes. However, no fluorescence signal was detected regardless of histological enhancement (S1A and S1B Fig).

**Fig 4:**
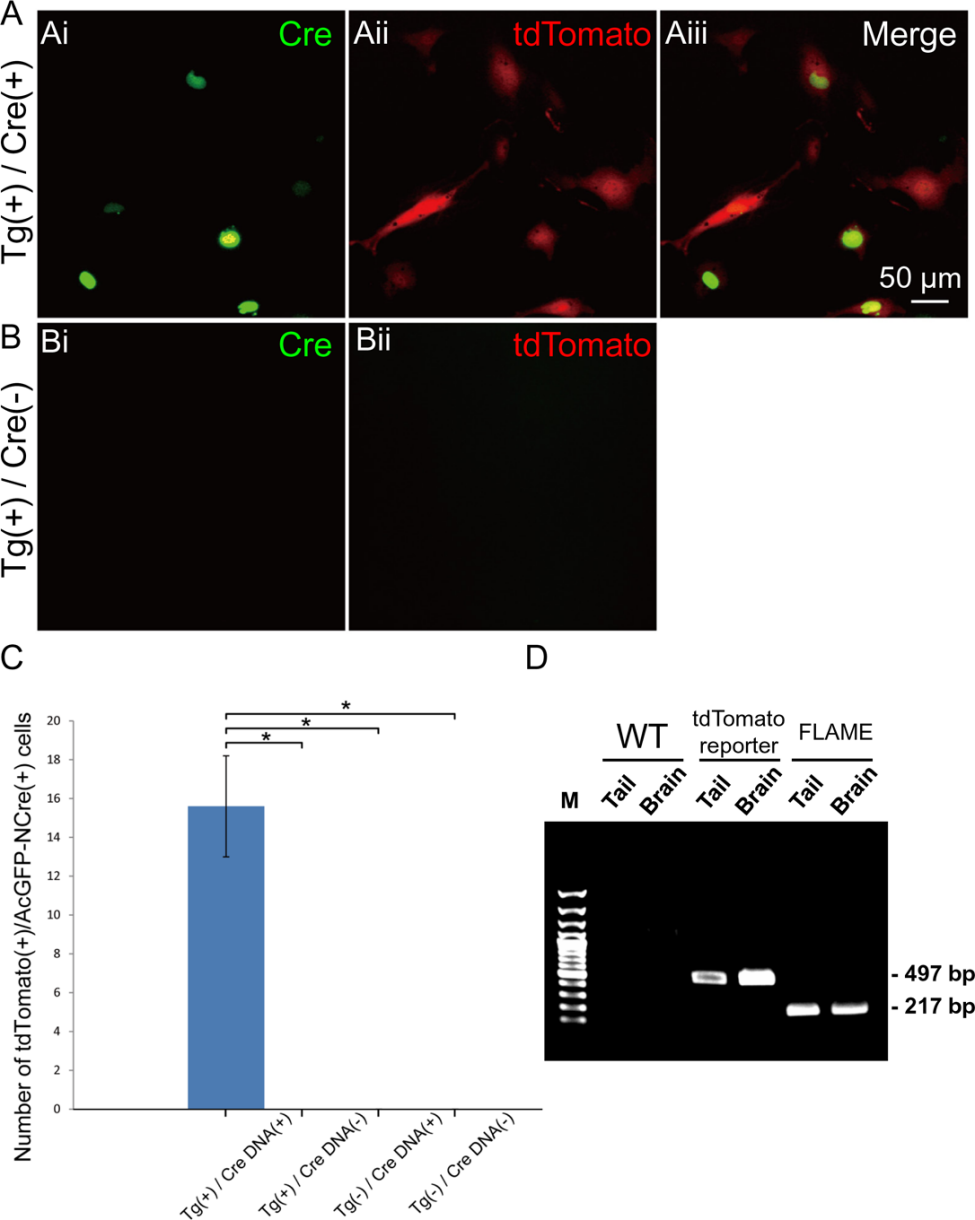
Reliability of the reporter system. (A) Primary fibroblasts prepared from the tail tip of the floxed STOP tdTomato reporter transgenic rats; the expressions of AcGFP-NCre by lipofection (i), tdTomato fluorescence (ii), and merged images (iii) are shown. (Bi–ii) Primary fibroblasts cultured from Tg-positive rats treated with vehicle alone. (C) Comparison of the ratio of tdTomato/AcGFP double-positive cells in each group (Tg, *n* = 5; wild-type, *n* = 6). Ten fields of interest were unintentionally chosen from each well, and double-blinded counting was applied to detect the fluorescent double-positive cells. In total 219 double positive cells were detected in group (A). *, *p* < 0.01, one-way ANOVA with Scheffe’s multiple comparison test. (D) PCR assay of the genomic DNA each extracted from the tail and the brain of a wild-type control rat (left 2 lanes), a tdTomato reporter rat (middle 2 lanes) or a FLAME (right 2 lanes). The allele of BAC with STOP sequence was detectable as 497 bp band whereas that without STOP as 217 bp band.

The leak of expression triggered by unexpected STOP excision can sometimes cause disturbances in loxP-based reporter animals [23,24]. To validate Cre-dependent expression in this rat line, primary cultured fibroblasts were transfected with the expression plasmids of *AcGFP-NCre*. Only fibroblasts with AcGFP-NCre–positive nuclei exhibited tdTomato in the cell soma (Fig 4Ai–4Aiii). On the other hand, no cells expressed tdTomato without *AcGFP-NCre* transfection (Fig 4Bi and 4Bii). As shown in Fig 4C, the leaked tdTomato expression was negligible in the absence of Cre, whereas the case-sensitive expression manifested in the presence of Cre. The robustness of reporter system was complemented further by PCR-based assay (Fig 4D). DNA templates were extracted from either tail or cortico-hippocampal tissue in the brain, and each allele in the BAC construct with (497 bp) or without STOP sequence (217 bp) was amplified by PCR. Only a single band corresponding to the allele with STOP was detected in the tdTomato reporter rat, suggesting that the background recombination is negligible. In the subsequent experiments, *Cre* was delivered to a subpopulation of neurons in each NBRP-0734 by transfection through *in utero* electroporation and crossbreeding with a *Cre*-driver rat.

### Spatiotemporal control of reporter gene expression by electroporation

**Fig 5:**
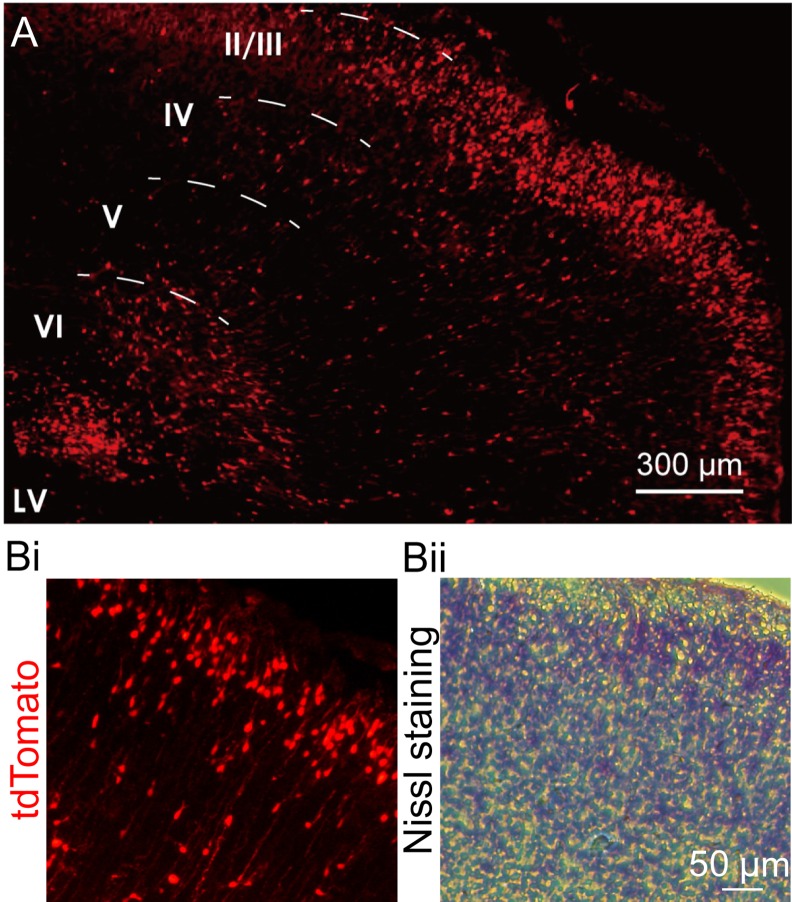
*In utero* electroporation of *Cre* plasmid in the brains of floxed STOP tdTomato reporter transgenic rats. (A) tdTomato expression in layer 2/3 of the cerebral cortex (P0, *n* = 2). The migrating neurons and L2/3 small pyramidal cells were fluorescently labeled with their axonal branches. LV: lateral ventricle. (**B**) Layer 2/3 neurons: tdTomato fluorescence (i) and Nissl staining (ii).

Plasmids containing *Cre* cDNA construct were injected into the left lateral ventricle of embryos at 18 days of gestation (E18) and transfected in the neuroblasts according to the *in utero* electroporation protocol [25]. In the NBPR-0734 heterozygotes with the floxed STOP tdTomato allele, tdTomato-positive neurons were mostly present in layer 2/3 of the left primary somatosensory cortex at P0–P1 (*n* = 2, Fig 5A and 5B). Some migrating neurons also expressed tdTomato. On the other hand, tdTomato fluorescence was negligible in the cortex of the wild-type rat.

### Directive expression using *Cre*-driver rats

**Fig 6:**
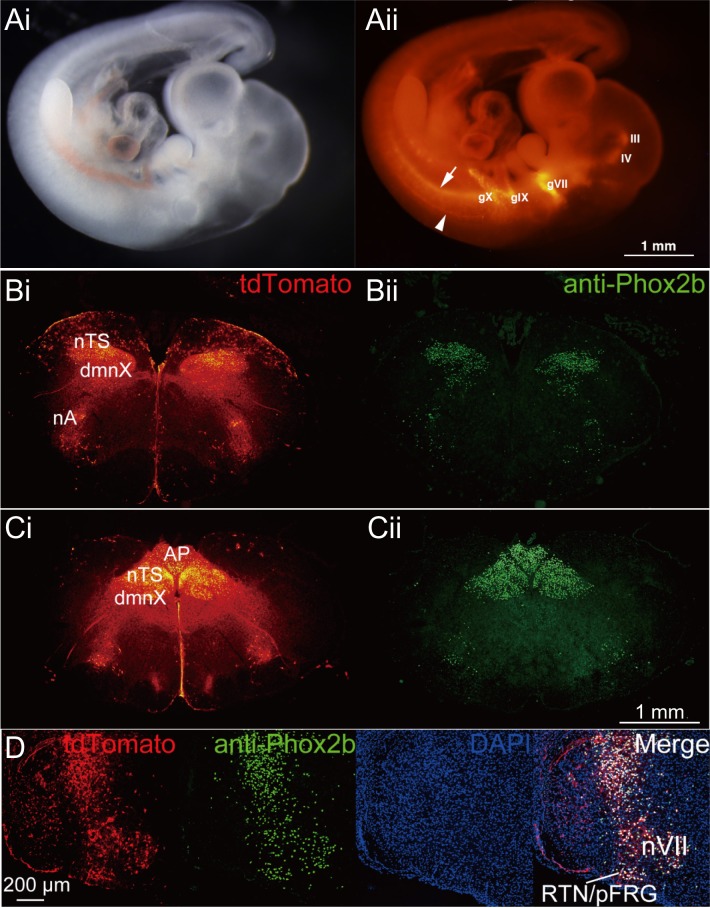
Specific expression of tdTomato in phox2b-Cre/floxed STOP tdTomato double transgenic rats. (A) Plain image (i) and tdTomato fluorescence image (ii) from whole embryos (E12.5): the oculomotor (III) and trochlear (IV) motor nuclei in the central nervous system (III and IV), and the three epibranchial sensory/distal ganglia; geniculate VII, petrosal IX, and nodose X ganglia (gVII, gIX, and gX, respectively). Note that the axons of spinal accessory nucleus (arrowhead) and neurons in the ventral neural tubes (arrow) are also fluorescent. (B, C) Brainstem of neonatal rat: tdTomato (i) and anti-Phox2b (ii). nTS, sensory neurons of the nucleus of the solitary tract; dmnX, dorsal motor nucleus of the vagus nerve; nA, nucleus ambiguus; AP, area postrema; VII, facial nucleus; RTN/pFRG, retrotrapezoid nucleus/parafacial respiratory group. (D) Co-localization of tdTomato and Phox2b: from left to right, tdTomato, anti-Phox2b, DAPI, and the merge.

A paired-like homeobox encoding gene (*Phox2b*) is a key regulator of autonomic neural crest derivatives and placode-derived visceral sensory ganglia. *Phox2b* is also expressed in pre-inspiratory neurons in the parafacial respiratory group, which constitute one of the respiratory rhythm generators in the medulla of newborn rats [26,27]. We recently generated a BAC *phox2b*-*Cre* driver rat to study respiratory rhythm generation and congenital central hypoventilation syndrome [28]. Double-Tg rats were obtained by crossbreeding BAC *phox2b*-*Cre* driver rats with the reporter rats, NBRP-0734. The embryos developed normally and expressed tdTomato signals in the epibranchial motor nuclei such as those forming the oculomotor (III), trochlear (IV), facial (VII), glossopharyngeal (IX), and vagal (X) nuclei as well as the sympathetic chain precursors at E12.5 (Fig 6Ai and 6Aii). This expression pattern is consistent with the distribution of endogenous Phox2b in embryonic mice [29] and rats [28]. In the brains of newborn rats, tdTomato signal was detected in the parafacial respiratory group complex in the medulla, where the endogenous Phox2b immunoreactivities were localized (Fig 6B–6D). Furthermore, the axonal trajectories were clearly traced by tdTomato fluorescence.

Hence, we established a tdTomato reporter system in rats that conditionally expresses tdTomato under the Cre/loxP system.

### Generation of FLAME strains

**Fig 7:**
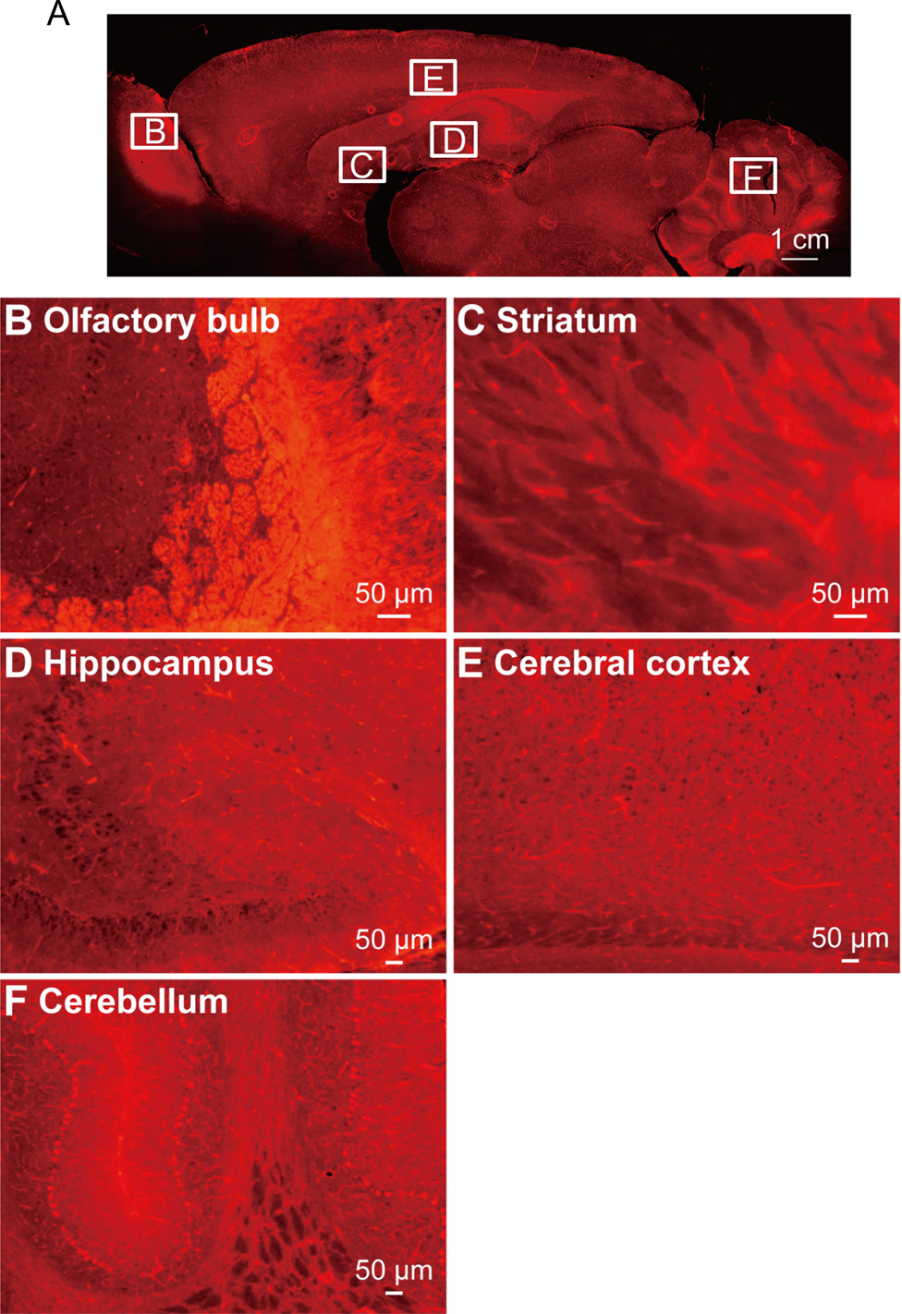
tdTomato expression in the brain of FLAME. (A) A sagittal section of the brain dissected from adult FLAME (n = 2). tdTomato was expressed ubiquitously in neurons throughout the brain, such as those in the olfactory bulb (B), the striatum (C), the hippocampus (D), the cerebral cortex (E), and the cerebellum (F).

**Fig 8:**
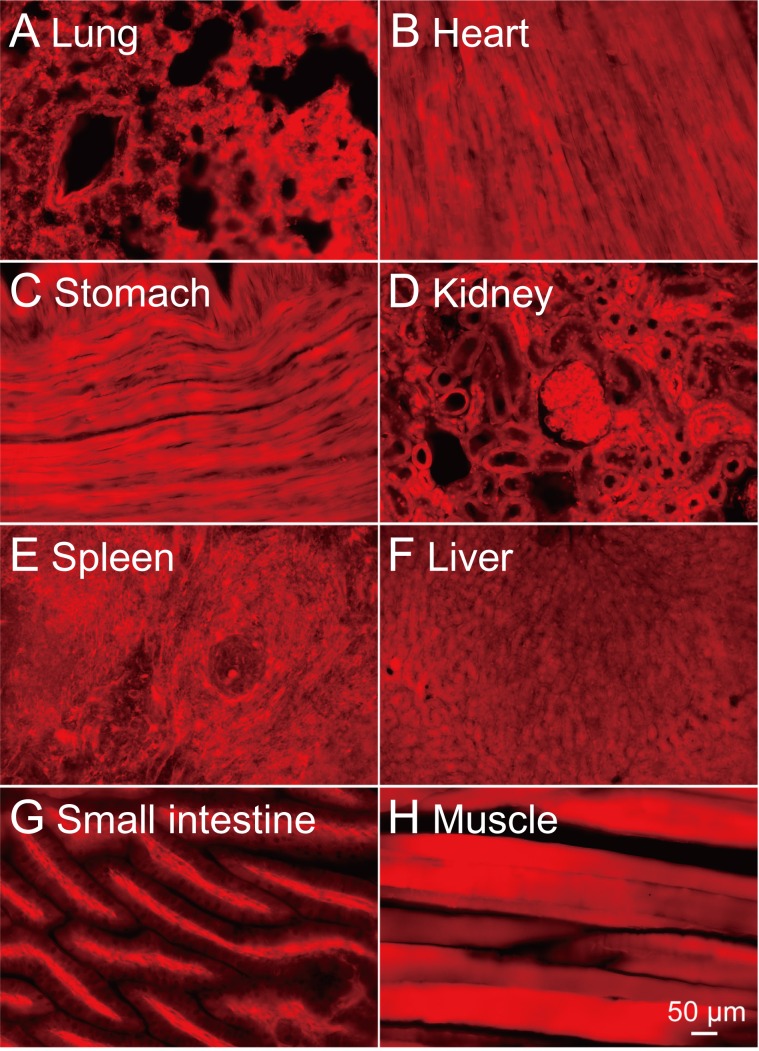
Ubiquitous and strong expression of tdTomato in major organs of FLAME. Bright-field images (left) and fluorescent images (right) of tissue sections (20 μm) prepared from lungs (A), a heart (B), a stomach (C), kidneys (D), a spleen (E), a liver (F), a small intestine (G), and a muscle (H). The scale in (H, 50 μm) is also applied to (A)-(G).

In order to verify the effectiveness of the tdTomato reporter system in other organs, we injected *Cre* mRNA into the fertilized ova of NBRP-0734 and established two stable lines that ubiquitously express tdTomato. Newborn pups of either line expressing tdTomato were clearly distinguishable from the null siblings by the bright red fluorescence from the skin under green excitation light (S2A Fig, left and right panels). Therefore, we nicknamed these strains “FLAME” (S3 Video). Indeed, only a single band corresponding to the allele without STOP (217 bp) was detected in either FLAME by the PCR assay of genomic DNA (Fig 4D). When the FLAME pups were dissected (*n* = 2, one from NBRP-0789 and another from -0790), all organs (e.g., the brain, lungs, stomach, heart, liver, spleen, and intestine) fluoresced bright red with tdTomato (S2B Fig, left and right panels, and S3 Fig) because almost all cells in the brain (Fig 7) and other tissues (Fig 8) were expressing tdTomato. Such cells as ova, primary macrophages, and red blood cells were also blightly fluoresced in red (S4A–S4C Fig, left and right panels). These results strongly suggest that the tdTomato reporter system effectively functions in various organs and tissues. No difference was found between two strains.

## Discussion

The present results show efficient and sensitive Cre-mediated reporter expression in Tg rats with an improved STOP cassette that comprises four successive polyadenylation signals inserted into the reporter genome. The strict regulation of tdTomato expression by Cre recombinase was evidenced by the following findings: (1) tdTomato was expressed preferentially in neurons transfected with *Cre* via AAV2 vectors; (2) tdTomato was expressed in cultured fibroblasts only when transfected with *Cre* gene; and (3) tdTomato was expressed only in neurons expressing Cre under a cell type–specific promotor using the BAC *Phox2b*-*Cre* driver rat. Therefore, any cells with a specified genetic trait can be selectively and reliably labelled with bright tdTomato fluorescence by combining our reporter rat with an appropriate driver rat.

Until now, a floxed STOP LacZ reporter rat was the only relevant line that adopted a Cre/loxP recombination system [30]. However, its application to living tissues and animals or for the three-dimensional imaging of relatively large tissue volumes is challenging, because histochemical processing is essential to visualize the Cre-expressing cells in sections. In contrast, in our reporter rat, Cre-positive neurons were visualized in the living slice or in the whole embryo three-dimensionally without any enhancement. Thus, the imaging of detailed structures such as the spine morphology of neurons deep in the tissue of a live animal is possible with the combination of advanced optics such as two-photon microscopy and light-sheet microscopy. Hence, the findings of the present study will strongly aid the generation of a variety of driver rats, which will facilitate physiological and anatomical studies using rats as an ideal model systems for human biology and disease [31].

Furthermore, our rat reporter system will facilitate connectome studies tracing the axonal projections of identified neurons, especially under *in vivo* image analysis, because of the abovementioned advantages as well as the strength of rat such as the relatively large body size and complex behavior. The bright tdTomato fluorescence would enable the visualization of the cells for long periods both *in vivo* and *ex vivo*; examples include tracing specific cell lineages and evaluating changes of the dendritic morphology of neurons. Flame rats, the new rat line derived from our tdTomato reporter strain, may also be an ideal tool for organ transplantation research owing to improved traceability of cells and tissues [6,9–11,22,32–34]. Furthermore, any tdTomato-labeled cells from Flame rats would be traceable in the host tissue, enabling visualization of how transplanted cells/tissues behave and interact with the host.

## Supporting Information

S1 Fig Dependence of tdTomato expression in the combination of AAV2-Cre and the reporter rat–control experiments. (A) Representative image of phosphate-buffered saline injection into the hippocampus of transgenic rats (*n* = 7). Untreated brain slices were directly examined by fluorescence microscopy. Injection of phosphate-buffered saline alone did not induce tdTomato expression. (B) Wild-type rats were injected with AAV2-Cre into the striatum (*n* = 2). AAV2-Cre did not exhibit red fluorescence, and tdTomato expression could not be detected even after immunohistochemical enhancement although Cre immunoreactivity was scattered as nucleus-like structures in the injection site.
(PDF)

S2 Fig Generation of FLAME. (A) P4 littermates obtained by crossing FLAME males with wild-type LE females: bright-field image (i) and fluorescent image (ii). Two of them (arrows) exhibited strong red fluorescence, whereas their siblings not. (B) Gross aspect of internal organs: bright-field image (i) and fluorescent image (ii).
(PDF)

S3 Fig Ubiquitous and strong expression of tdTomato in FLAME. Isolated major organs. Bright-field images (left) and fluorescent images (right) of the brain, stomach and liver, lungs, spleen, heart, and intestine are shown.
(PDF)

S4 Fig tdTomato expression in ova, abdominal macrophages, and red blood cells. (A) Ova were collected through super ovulation. All collected ova exhibited fluorescence. Follicle cells surrounding the ova also strongly expressed tdTomato. tdTomato expression was also observed in both abdominal macrophages (B) and red blood cells (C). Cells could be identified by excitation light exposure.
(PDF)

S1 Video Acquired coronal images were reconstructed in 3D. Related to Fig 3. Scale bar: 50 μm.
(AVI)

S2 Video Reconstructed 3D image of CUBIC-treated hippocampal tissue. Rostral to caudal direction. Related to Fig 3. Scale bar: 50 μm.
(AVI)

S3 Video Fluorescent image of FLAME pup. Newborn pup (P5) was placed under the excitation light illuminator (510 nm). Whole body emitted strong red fluorescence.
(MP4)
