# Supplementary material for: A Novel Reporter Rat Strain That Conditionally Expresses the Bright Red Fluorescent Protein tdTomato
Source: PLoS One. 2016 May 19;11(5):e0155687. doi: 10.1371/journal.pone.0155687 (PMC4873025; doi:10.1371/journal.pone.0155687)
Supplement: S1 Fig — (A) Representative image of phosphate-buffered saline injection into the hippocampus of transgenic rats (n = 7). Untreated brain slices were directly examined by fluorescence microscopy. Injection of phosphate-buffered saline alone did not induce tdTomato expression. (B) Wild-type rats were injected with AAV2-Cre into the striatum (n = 2). AAV2-Cre did not exhibit red fluorescence, and tdTomato expression could not be detected even after immunohistochemical enhancement although Cre immunoreactivity was scattered as nucleus-like structures in the injection site. (PDF) [file pone.0155687.s001.pdf]

S1 Figure

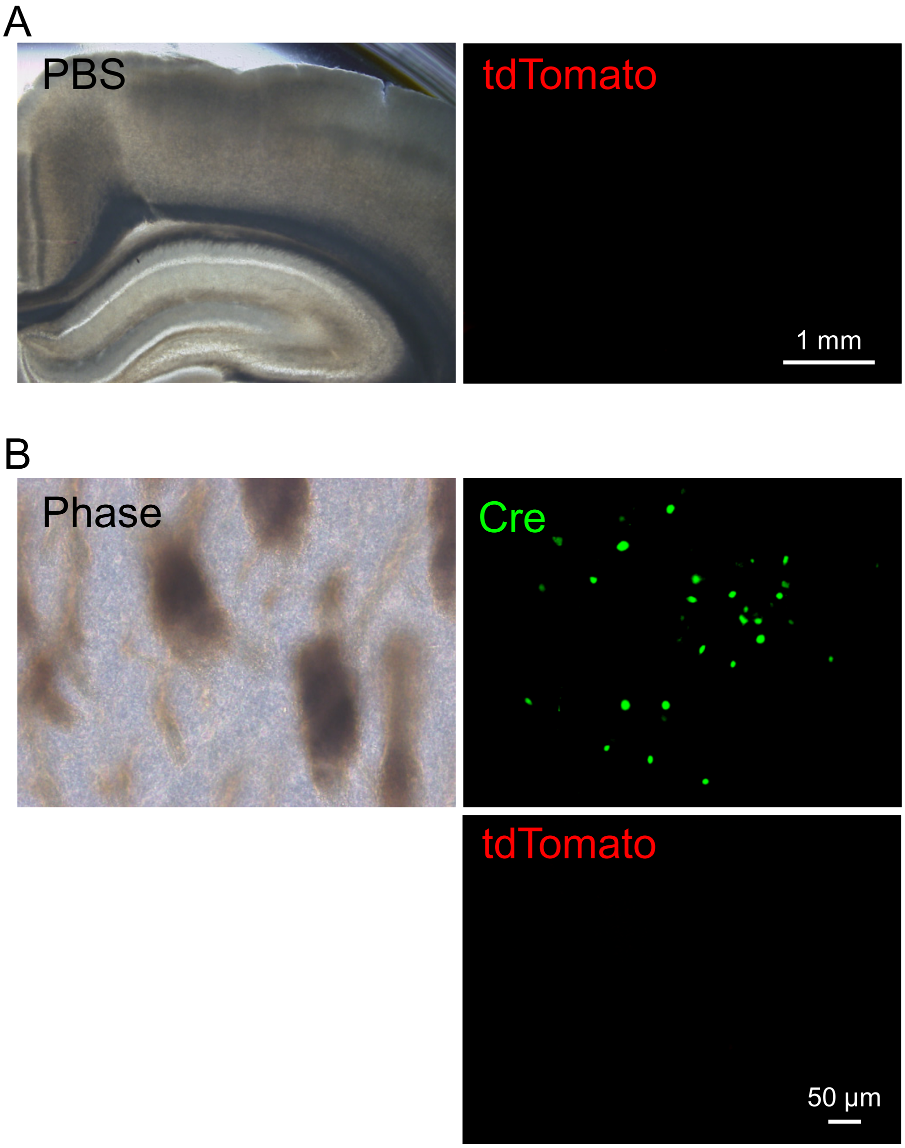

S1 Fig. Dependence of tdTomato expression in the combination of AAV2-Cre and the reporter rat – control experiments.
(A) Representative image of phosphate-buffered saline injection into the hippocampus of transgenic rats (n = 7). Untreated brain slices were directly examined by fluorescence microscopy. Injection of phosphate-buffered saline alone did not induce tdTomato expression. (B) Wild-type rats were injected with AAV2-Cre into the striatum (n = 2). AAV2-Cre did not exhibit red fluorescence, and tdTomato expression could not be detected even after immunohistochemical enhancement although Cre immunoreactivity was scattered as nucleus-like structures in the injection site.
