# Supplementary material for: A Novel Reporter Rat Strain That Conditionally Expresses the Bright Red Fluorescent Protein tdTomato
Source: PLoS One. 2016 May 19;11(5):e0155687. doi: 10.1371/journal.pone.0155687 (PMC4873025; doi:10.1371/journal.pone.0155687)
Supplement: S2 Fig — (A) P4 littermates obtained by crossing FLAME males with wild-type LE females: bright-field image (i) and fluorescent image (ii). Two of them (arrows) exhibited strong red fluorescence, whereas their siblings not. (B) Gross aspect of internal organs: bright-field image (i) and fluorescent image (ii). (PDF) [file pone.0155687.s002.pdf]

S2 Figure

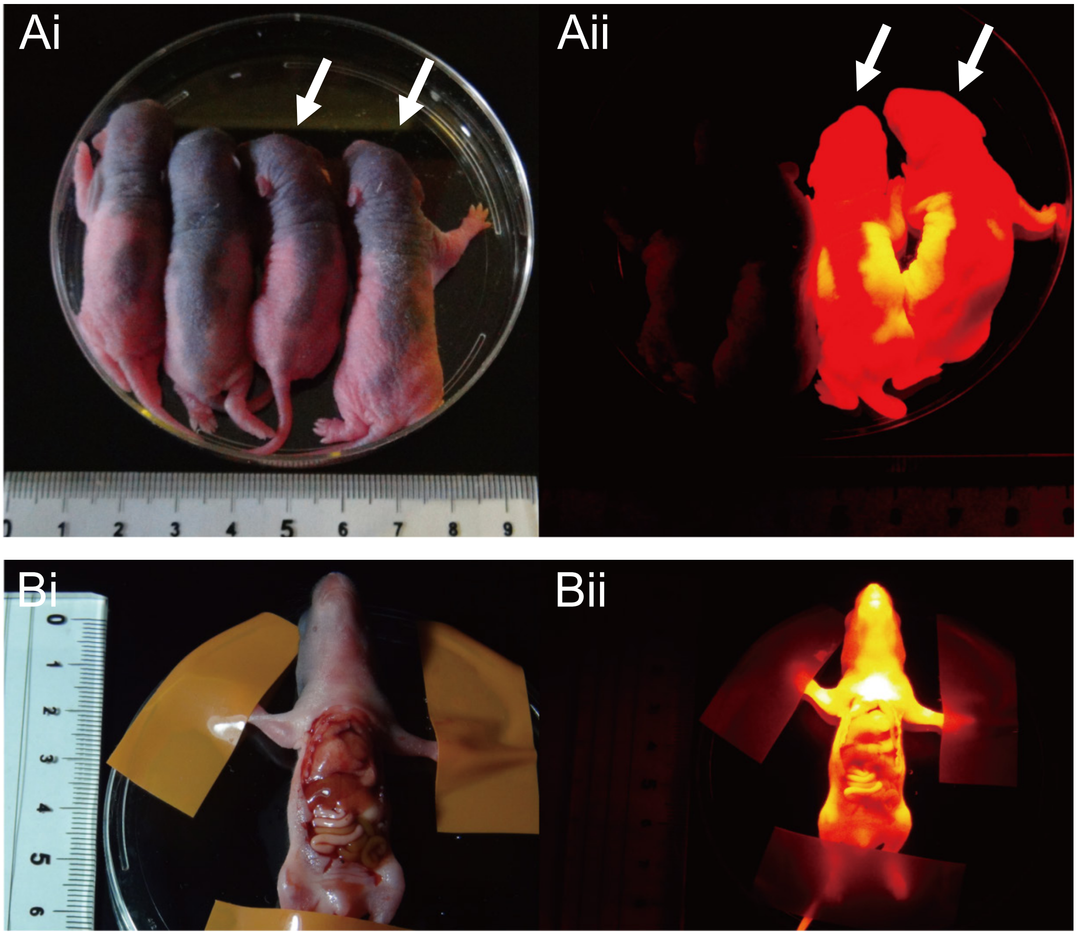

S2 Fig. Generation of FLAME.
(A) P4 littermates obtained by crossing FLAME males with wild-type LE females: bright-field image (i) and fluorescent image (ii). Two of them (arrows) exhibited strong red fluorescence, whereas their siblings not. The scale is shown in (i). (B) Gross aspect of internal organs: bright-field image (i) and fluorescent image (ii). The scale is shown in (i).
