# Supplementary material for: A Novel Reporter Rat Strain That Conditionally Expresses the Bright Red Fluorescent Protein tdTomato
Source: PLoS One. 2016 May 19;11(5):e0155687. doi: 10.1371/journal.pone.0155687 (PMC4873025; doi:10.1371/journal.pone.0155687)
Supplement: S3 Fig — Isolated major organs. Bright-field images (left) and fluorescent images (right) of the brain, stomach and liver, lungs, spleen, heart, and intestine are shown. (PDF) [file pone.0155687.s003.pdf]

S3 Figure

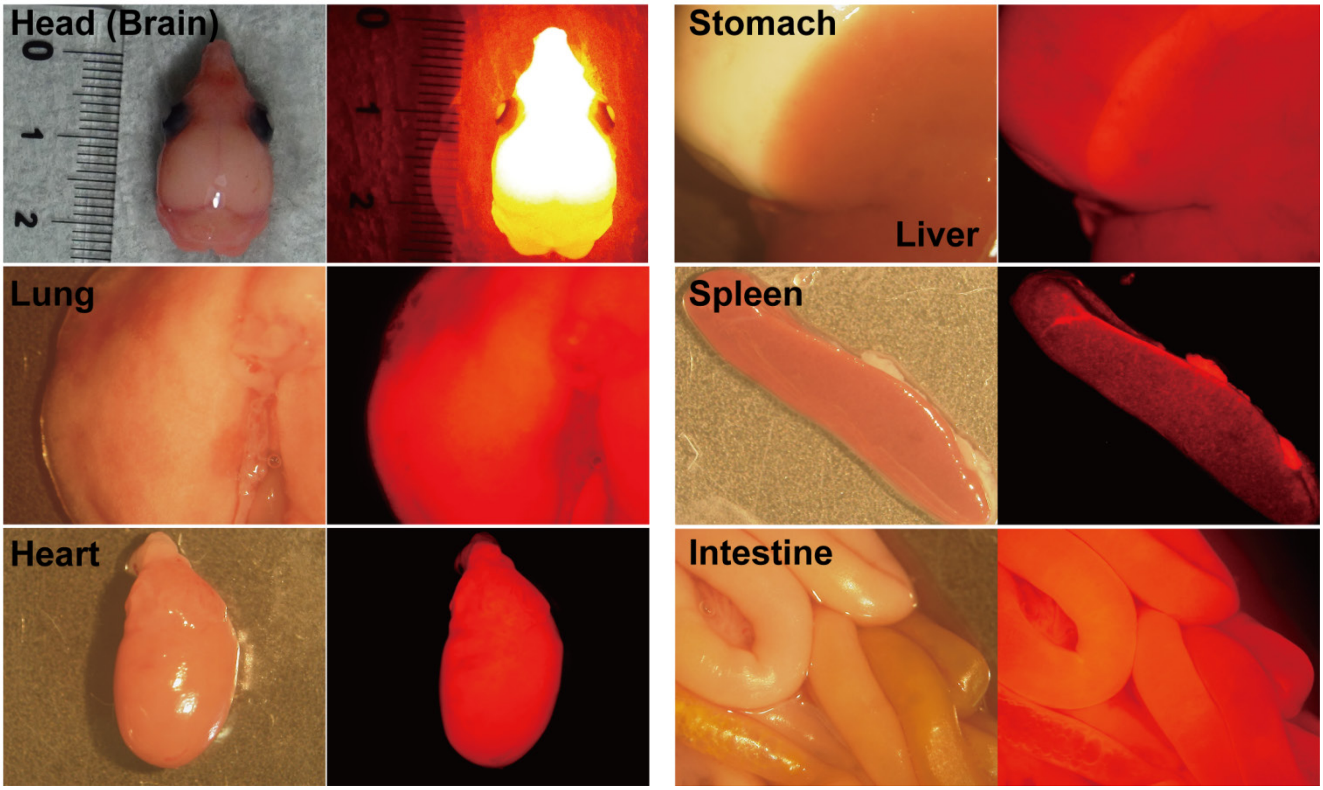

S3 Fig. Ubiquitous and strong expression of tdTomato in FLAME.
Isolated major organs. Bright-field images (left) and fluorescent images (right) of the brain, stomach and liver, lungs, spleen, heart, and intestine are shown.
