# Supplementary material for: A Novel Reporter Rat Strain That Conditionally Expresses the Bright Red Fluorescent Protein tdTomato
Source: PLoS One. 2016 May 19;11(5):e0155687. doi: 10.1371/journal.pone.0155687 (PMC4873025; doi:10.1371/journal.pone.0155687)
Supplement: S4 Fig — (A) Ova were collected through super ovulation. All collected ova exhibited fluorescence. Follicle cells surrounding the ova also strongly expressed tdTomato. tdTomato expression was also observed in both abdominal macrophages (B) and red blood cells (C). Cells could be identified by excitation light exposure. (PDF) [file pone.0155687.s004.pdf]

# S4 Figure

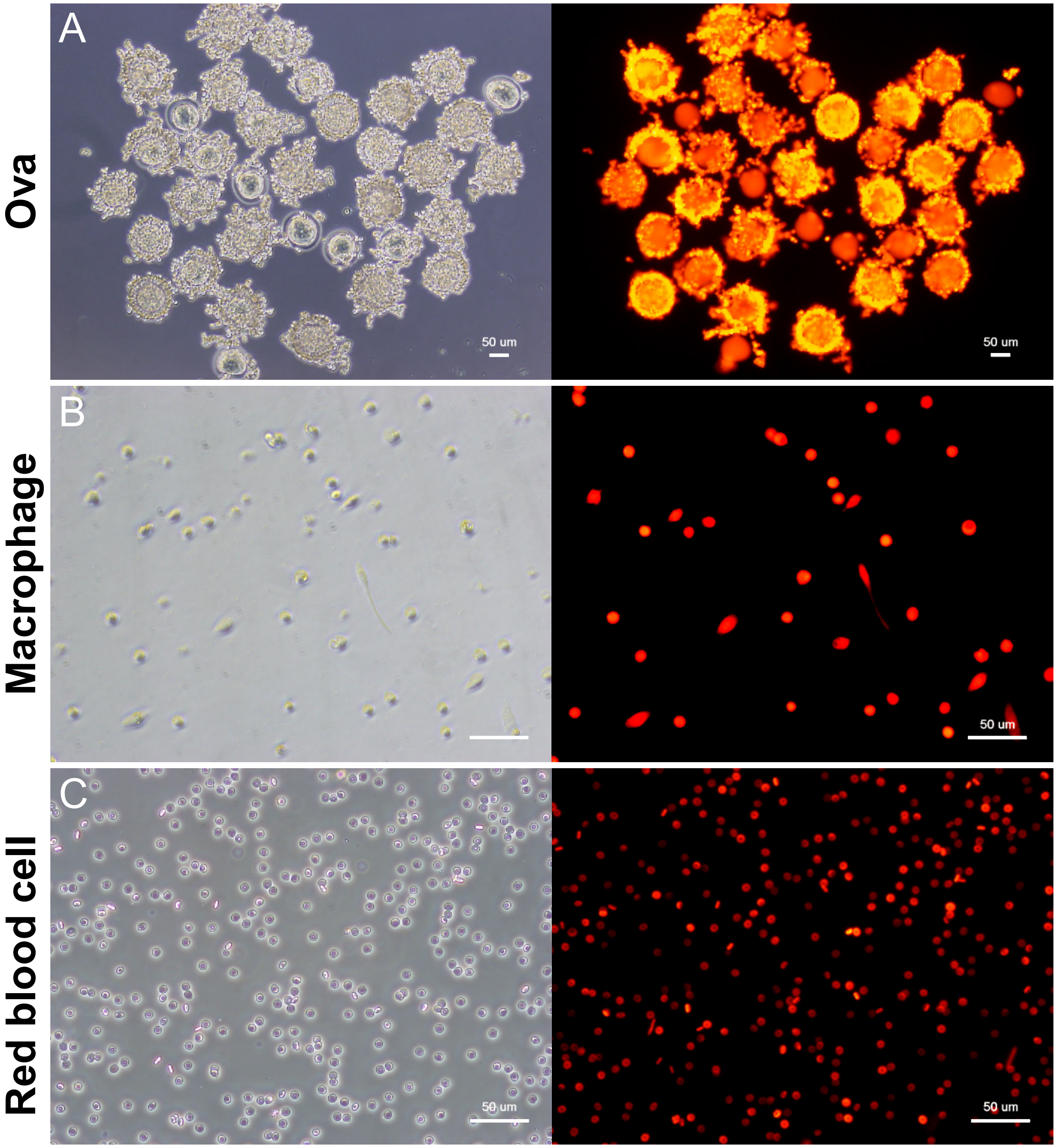


**S4 Fig. tdTomato expression in ova, abdominal macrophages, and red blood cells.** (A) Ova were collected through super ovulation. All collected ova exhibited fluorescence. Follicle cells surrounding the ova also strongly expressed tdTomato. tdTomato expression was also observed in both abdominal macrophages (B) and red blood cells (C). Cells could be identified by excitation light exposure. Scale bar, 50 μm for each.
